# Supplementary material for: Tracing the first hematopoietic stem cell generation in human embryo by single-cell RNA sequencing
Source: Cell Res. 2019 Sep 9;29(11):881–94. doi: 10.1038/s41422-019-0228-6 (PMC6888893; doi:10.1038/s41422-019-0228-6)
Supplement: Supplementary file 1 — Supplementary Figure 1 [file 41422_2019_228_MOESM1_ESM.pdf]

a

Stage	Tissue	Sorting Marker	Sequencing Strategy	Cell Number	Cell Number after QC[d]
CS 10 (23 dpc)	Body	7-AAD$^-$	10X Genomics	6666	6662
CS 11 (24 dpc)	CH[a]	7-AAD$^-$		4186	4186
CS 13 (30 dpc)	DA[b]	7-AAD$^-$CD235a$^-$		592	592
Total				11444	11440
CS 12 (27 dpc)	CH	CD235a$^-$CD45$^-$CD34$^+$CD44$^+$	Modified STRT-Seq	96	95
		CD235a$^-$CD45$^-$CD34$^+$CD44$^-$		48	45
CS 13 (29 dpc)	AGM[c]	CD235a$^-$CD45$^-$CD34$^+$CD44$^+$		48	48
		CD235a$^-$CD45$^-$CD34$^+$CD44$^-$		48	48
CS 14 (32 dpc)	AGM	CD235a$^-$CD45$^-$CD34$^+$CD44$^+$		48	48
		CD235a$^-$CD45$^-$CD34$^+$CD44$^-$		48	46
CS 15 (36 dpc)	DA	CD235a$^-$CD45$^-$CD34$^+$		192	179
Total				528	509

[a]CH, caudal half; [b]DA, dorsal aorta; [c]AGM, aorta-gonad-mesonephros region; [d]QC: quality control

b

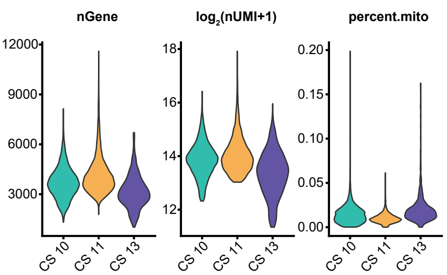

c

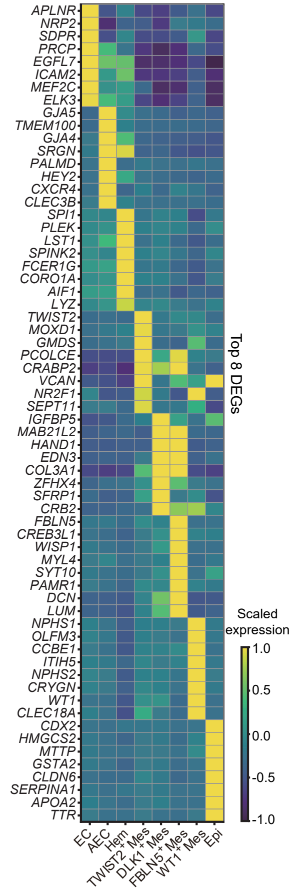

d

	GO terms	Adjusted *P* value
EC	vasculogenesis	2.381778e-10
	extracellular structure organization	7.475514e-09
	endothelial cell migration	1.242495e-08
	regulation of angiogenesis	1.701371e-08
AEC	regulation of vasculature development	2.918432e-09
	endothelium development	7.530313e-09
	artery development	7.034866e-07
	aorta development	1.653553e-06
Hem	protein targeting to ER	6.313973e-06
	regulation of lymphocyte proliferation	1.296278e-05
	leukocyte chemotaxis	1.333360e-05
	nuclear-transcribed mRNA catabolic process	1.617695e-05
TWIST2$^+$ Mes	ossification	1.931562e-05
	osteoblast differentiation	5.233404e-04
	muscle contraction	6.952311e-04
	connective tissue development	8.671836e-04
DLK1$^+$ Mes	muscle tissue development	0.0003054646
	anterior/posterior pattern specification	0.0003054646
	cardiac ventricle morphogenesis	0.0003373970
	cornification	0.0009012184
FBLN5$^+$ Mes	extracellular matrix organization	1.106941e-12
	mesenchyme development	5.203070e-08
	regulation of cell morphogenesis	7.441771e-07
	collagen fibril organization	7.441771e-07
WT1$^+$ Mes	nephron development	1.207785e-09
	renal system development	8.988228e-09
	kidney epithelium development	9.277739e-09
	cell differentiation involved in kidney development	4.839030e-08
Epi	regulation of cell-cell adhesion	1.270592e-05
	columnar/cuboidal epithelial cell differentiation	3.338444e-05
	regulation of cholesterol esterification	9.886685e-05
	plasma lipoprotein particle assembly	1.397566e-04

e

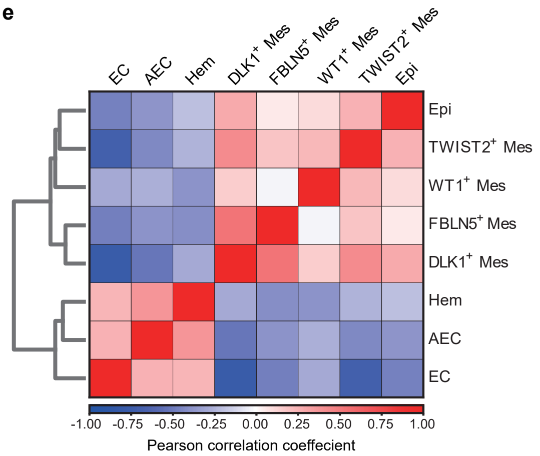

f

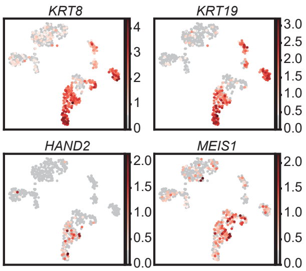

g

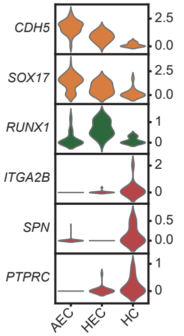

Supplementary Figure 1. Detailed feature of transcriptomically defined cell populations in CS 13 DA

a. Sample information for scRNA-seq data. **b**. Violin plots of cells from 10X-derived datasets (CS 10, CS 11 and CS 13) showing nGene, log_2(nUMI+1) and mitochondrial gene expression levels. **c**. Heatmap showing the average expressions of top 8 DEGs for eight clusters. **d**. The enriched major GO:BP terms for each cluster. **e**. Correlation and hierarchical clustering analyses of eight clusters showing that AEC and Hem clusters are more similar with each other, while four mesenchymal populations and Epi cluster correlate more closely to each other. **f**. UMAP plots showing the expression of several featured genes of stromal cells. **g**. Violin plots showing the expression of endothelial and hematopoietic genes in AEC, HEC and HC clusters.
