# Supplementary material for: Tracing the first hematopoietic stem cell generation in human embryo by single-cell RNA sequencing
Source: Cell Res. 2019 Sep 9;29(11):881–94. doi: 10.1038/s41422-019-0228-6 (PMC6888893; doi:10.1038/s41422-019-0228-6)
Supplement: Supplementary file 2 — Supplementary Figure 2 [file 41422_2019_228_MOESM2_ESM.pdf]

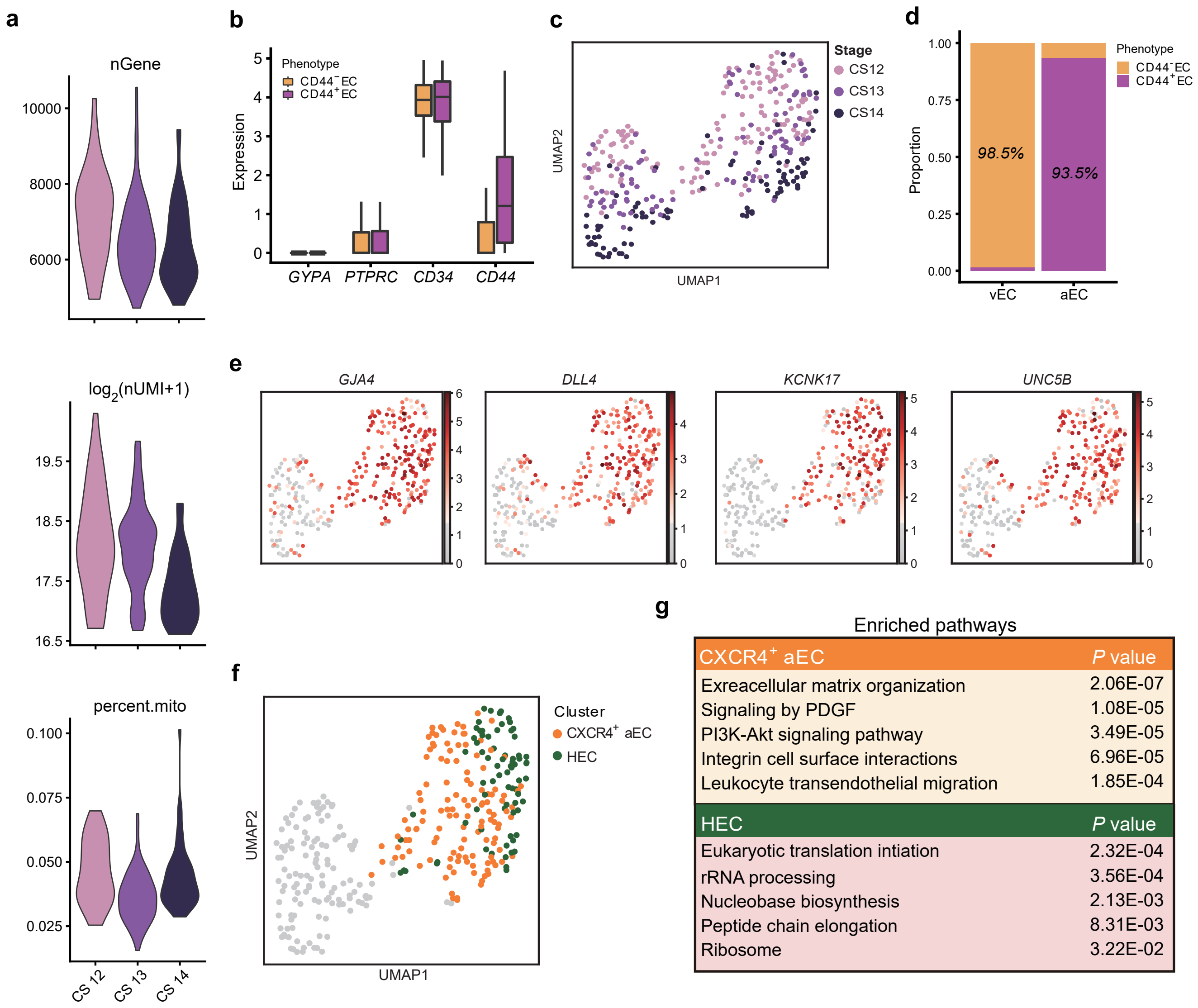

CXCR4⁺ aEC	*P* value
Exreacellular matrix organization	2.06E-07
Signaling by PDGF	1.08E-05
PI3K-Akt signaling pathway	3.49E-05
Integrin cell surface interactions	6.96E-05
Leukocyte transendothelial migration	1.85E-04
**HEC**	***P* value**
Eukaryotic translation intiation	2.32E-04
rRNA processing	3.56E-04
Nucleobase biosynthesis	2.13E-03
Peptide chain elongation	8.31E-03
Ribosome	3.22E-02

Supplementary Figure 2. Quality of scRNA-seq data of $CD44^+$/$CD44^-$ ECs and feature of two sub-divided aEC clusters

a. Violin plots displaying gene counts, log_2(nUMI+1) and mitochondrial gene percentages of scRNA-seq data generated from $CD44^+$/$CD44^-$ ECs of CS 12 CH, CS 13 and CS 14 AGM region. **b**. Box plots for the expression of surface markers used for FACS sorting. *GYPA* encodes CD235a and *PTPRC* encodes CD45. **c**. UMAP plot with different sampling stages mapped on it. **d**. Bar plots showing the proportion of immunophenotypic $CD44^+$ EC and $CD44^-$ EC in aEC and vEC clusters. **e**. UMAP plots showing the expression of several arterial genes. **f**. Mapping of $CXCR4^+$ aEC and HEC on the UMAP depicted in Fig. 2b. **g**. Pathways respectively enriched in two aEC sub-clusters.
