# Supplementary material for: Tracing the first hematopoietic stem cell generation in human embryo by single-cell RNA sequencing
Source: Cell Res. 2019 Sep 9;29(11):881–94. doi: 10.1038/s41422-019-0228-6 (PMC6888893; doi:10.1038/s41422-019-0228-6)
Supplement: Supplementary file 3 — Supplementary Figure 3 [file 41422_2019_228_MOESM3_ESM.pdf]

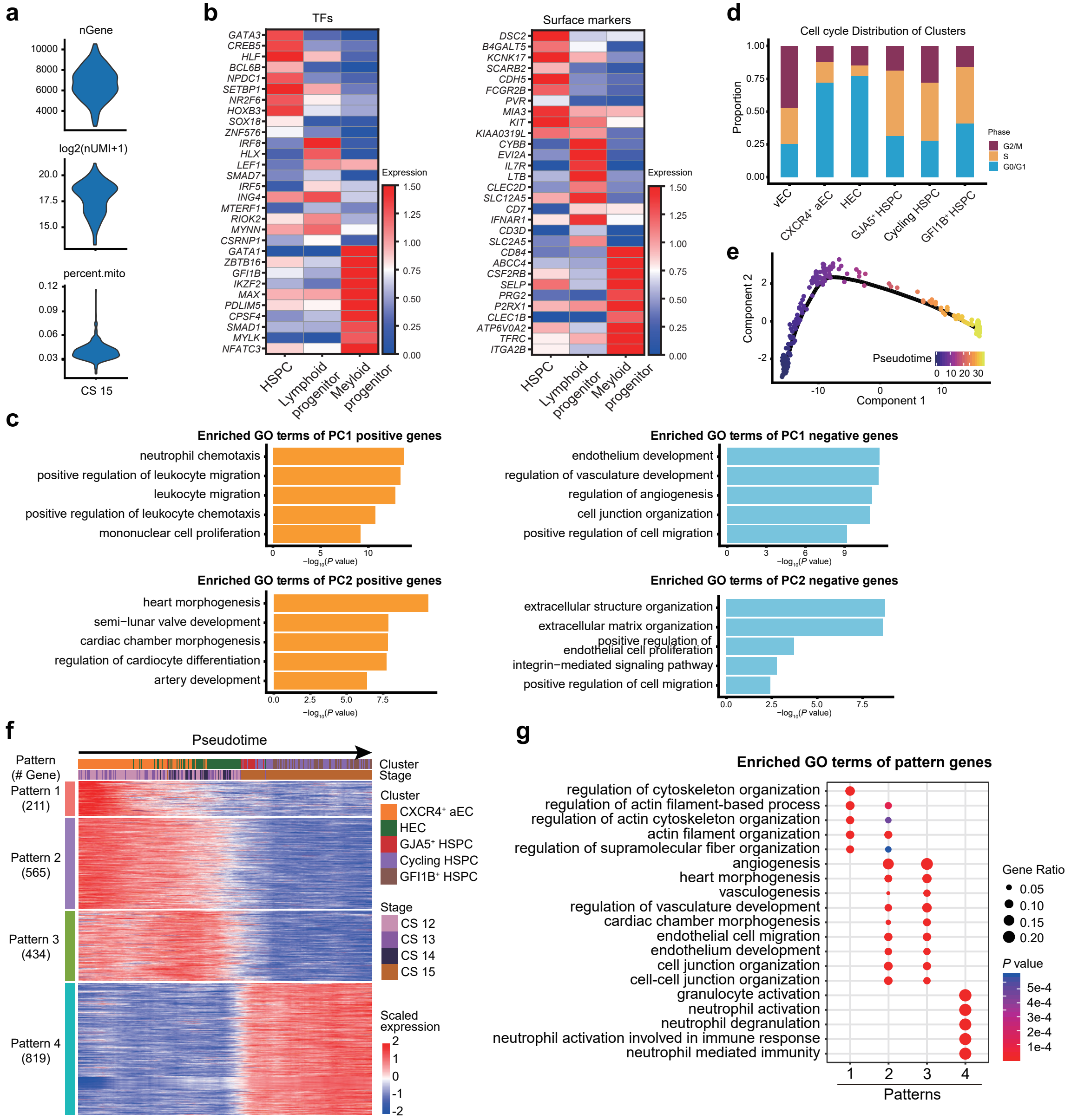

Supplementary Figure 3. Different gene expression patterns and cell cycle status of the cells along the endothelial-to-HSPC transition

a. Violin plots displaying gene counts, $\log_2$(nUMI+1) and mitochondrial gene percentages of scRNA-seq data generated from $CD45^{+}CD34^{+}$ cells of CS 15 DA. **b**. Heatmaps of the scaled average expressions of top 10 differentially expressed TFs (left) and surface markers (right) in HSPC (HSPC1/2/3), Myeloid progenitor and Lymphoid progenitor clusters. **c**. The enriched GO:BP terms for PC 1 (upper) and PC 2 (lower) positive (left) and negative (right) genes from PCA shown in Fig. 3f. **d**. Cell cycle distribution in vEC, $CXCR4^{+}$ aEC, HEC and three HSPC clusters ($GJA5^{+}$ HSPC, Cycling HSPC and $GFI1B^{+}$ HSPC). **e**. Pseudotime analysis by Monocle 2. **f**. Heatmap showing the scaled expressions of pattern genes along the trajectory axis inferred by Monocle 2 (smoothed over 20 adjacent cells). Note four patterns are revealed with the number of genes of each pattern is shown. **g**. The enriched GO terms of genes of four different expression patterns.
