# Supplementary material for: Tracing the first hematopoietic stem cell generation in human embryo by single-cell RNA sequencing
Source: Cell Res. 2019 Sep 9;29(11):881–94. doi: 10.1038/s41422-019-0228-6 (PMC6888893; doi:10.1038/s41422-019-0228-6)
Supplement: Supplementary file 4 — Supplementary Figure 4 [file 41422_2019_228_MOESM4_ESM.pdf]

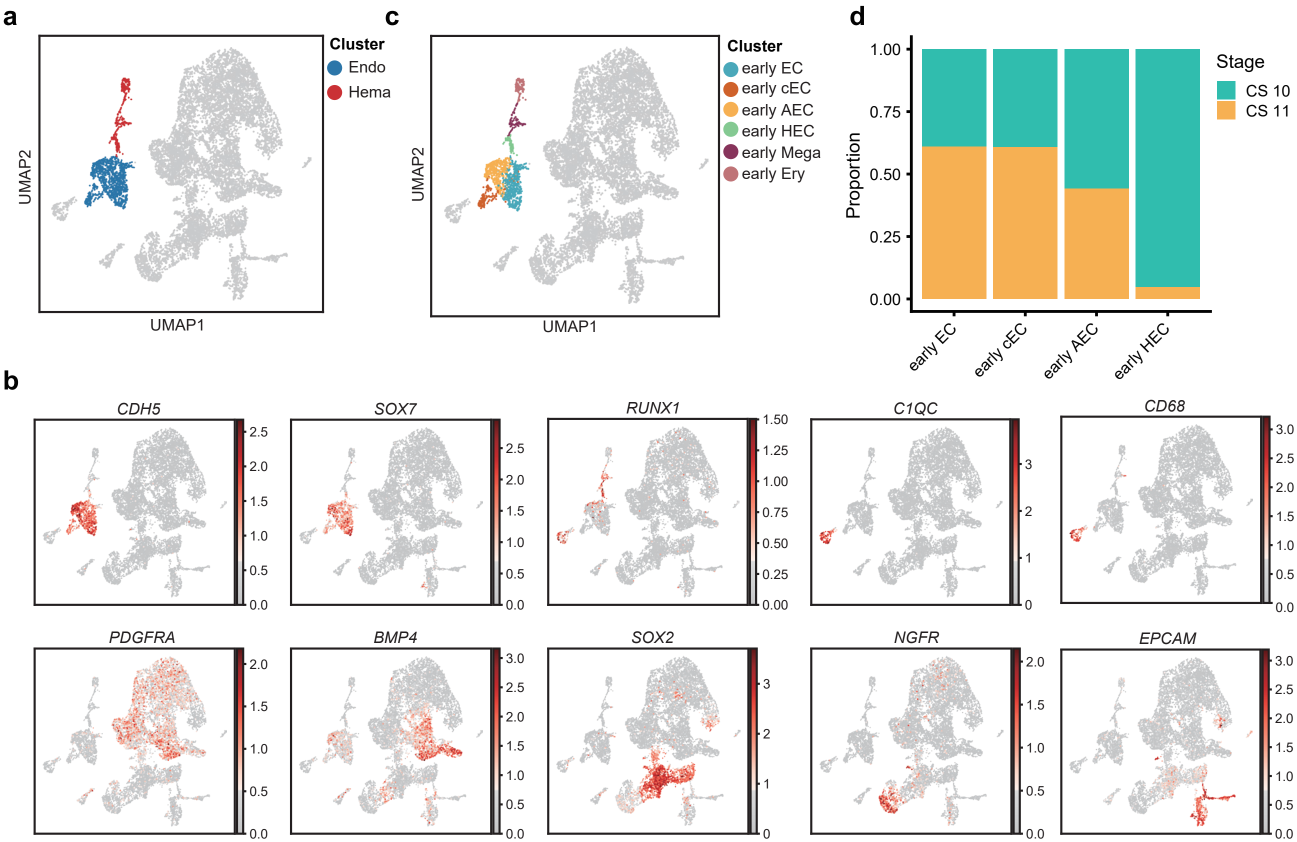

Supplementary Figure 4. Identification of hemogenic EC-related populations in early stages (CS 10 and CS 11) **a**. Mapping of endothelial (Endo) and hematopoietic cells (Hema) in CS 10 and CS 11. Other populations are shown in gray. **b**. The expression of endothelial, hematopoietic and mesenchymal genes that can help to identify different cell populations. **c**. UMAP with the sub-divided clusters shown in Fig. 4a mapped on it. **d**. Bar plots showing cellular constitutions of two sampling stages in the EC populations depicted in Fig. 4a.
