# Supplementary material for: Tracing the first hematopoietic stem cell generation in human embryo by single-cell RNA sequencing
Source: Cell Res. 2019 Sep 9;29(11):881–94. doi: 10.1038/s41422-019-0228-6 (PMC6888893; doi:10.1038/s41422-019-0228-6)
Supplement: Supplementary file 5 — Supplementary Figure 5 [file 41422_2019_228_MOESM5_ESM.pdf]

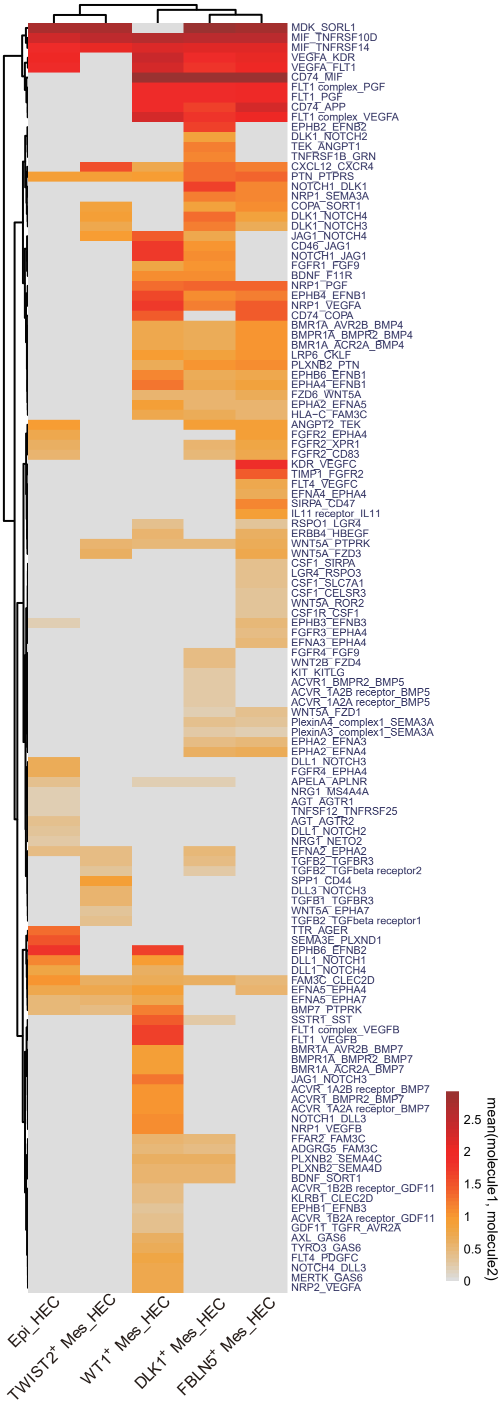

Supplementary Figure 5. The computationally predicted cell-cell interactions that potentially involved in the development of HSC-primed hemogenic ECs

Heatmap showing the scaled average expressions of ligand-receptor pairs in distinct stromal populations when coupled with HEC. Complete list and detailed ligand-receptor genes are shown. The hierarchical clustering result of cell populations (columns) and ligand-receptor pairs (rows) is shown.
